# The evolution of CpG density and lifespan in conserved primate and mammalian promoters

**DOI:** 10.18632/aging.101413

**Published:** 2018-04-14

**Authors:** Adam T. McLain, Christopher Faulk

**Affiliations:** 1 Department of Biology and Chemistry, College of Arts and Sciences, SUNY Polytechnic Institute, Utica, NY 13502, USA; 2 Department of Animal Sciences, University of Minnesota, College of Food, Agricultural, and Natural Resource Sciences, Saint Paul, MN 55108, USA

**Keywords:** evolution, CpG site, lifespan, CpG density, varmints, DNA methylation

## Abstract

Gene promoters are evolutionarily conserved across holozoans and enriched in CpG sites, the target for DNA methylation. As animals age, the epigenetic pattern of DNA methylation degrades, with highly methylated CpG sites gradually becoming demethylated while CpG islands increase in methylation. Across vertebrates, aging is a trait that varies among species. We used this variation to determine whether promoter CpG density correlates with species’ maximum lifespan. Human promoter sequences were used to identify conserved regions in 131 mammals and a subset of 28 primate genomes. We identified approximately 1000 gene promoters (5% of the total), that significantly correlated CpG density with lifespan. The correlations were performed via the phylogenetic least squares method to account for trait similarity by common descent using phylogenetic branch lengths. Gene set enrichment analysis revealed no significantly enriched pathways or processes, consistent with the hypothesis that aging is not under positive selection. However, within both mammals and primates, 95% of the promoters showed a positive correlation between increasing CpG density and species lifespan, and two thirds were shared between the primate subset and mammalian datasets. Thus, these genes may require greater buffering capacity against age-related dysregulation of DNA methylation in longer-lived species.

## Introduction

Gene promoters are conserved across the animal radiation and as far back as non-fungi eukaryotes (i.e. holozoans) [1]. Promoters have many features, including TATA boxes, DNA binding sites, and most notably for our investigation, enrichment in CpG sites, which in high enough density are designated as CpG islands (CGIs) [2]. Much evolutionary focus has been on pairwise comparison of promoters between human and mouse, or human and chicken [3]. Yet, questions remain about the evolution of promoter features that can only be answered by comparison of multiple genomes of animals. Here we use the Eukaryotic Promoter Database ‘new’ (EPDnew) repository of verified human promoters to serve as a base for identifying promoters in other species [4]. In this study, we compare a genomic feature, CpG site density in promoters and a physiological trait, maximum lifespan, in several dozen species.

Methylation of the cytosine in CpG dinucleotides results in the formation of 5-methylcytosine, a covalent modification that can impact gene expression. As such, CpGs are of particular interest for understanding gene function and the impact of epigenetic influences. Evolution can also be driven by methylation state. Methylated CpGs are prone to deamination and conversion to uracil, read as thymine by DNA polymerases, resulting in TpG mutations which rapidly deplete mammalian genomes of CpG sites over evolutionary time. For example, the average percentage of nucleotide substitutions between human and chimpanzee is 0.92% while at CpG sites, the rate rises to 15.2% [5]. However, CpGs located in CGIs are highly conserved across vertebrate genomes [6]. Despite the fact that methylated CpGs are the most rapidly mutating dinucleotides in the genome, Hartono et al. observed a high level of conservation of CpGs located within the promoter regions of genes highly conserved across 60 chordate genomes [7]. Previously we have shown methylation level can be conserved for hundreds of millions of years, at least in ultraconserved genes [8]. Generally, CpGs located within CGI promoters are hypomethylated, a feature conserved across the vertebrate radiation [9,10].

As vertebrates age, the epigenomic pattern of DNA methylation degrades, with the highly methylated CpG sites gradually becoming demethylated, while CGIs increase in methylation [11]. Therefore, DNA methylation becomes dysregulated as a function of aging and high CpG density may delay or buffer specific regions from age-related changes. Some gene exons have undergone accelerated evolution in long-lived species as their protein function is under selection [12,13]. However, unlike coding sequences, promoter regions alter gene expression, not protein function, so different species can regulate expression without altering the protein function. Within promoter regions the rapid mutation of CpG sites and their function in epigenetic gene expression make them prime targets for natural selection. We chose CpG site density because density alone is sufficient to predict methylation level [14]. Since methylation degrades over an individual's lifespan, we reasoned that selection for long lifespan may act not only on gene coding regions but on promoter regions. This selection would change promoter CpG density for genes whose expression must be more tightly regulated to allow for longer lifespan.

Across vertebrates, aging is a trait that varies among species. Despite the broad difference in lifespan between mice and humans (~2 years vs. ~90 years respectively), epigenetic clocks have been developed to determine biological age [15]. In order to see patterns in gradual genetic change compared to a gradation of lifespans in different species, we used the AnAge database of aging and longevity. It is a comprehensive resource developed for comparative biology studies, containing life history traits of over 4000 species [16].

To determine statistically how similar traits are across species despite independent evolutionary pressure, we must account for species’ relatedness, whereas ordinary statistical tests assume no relation. A barrier to simple correlation between physiological features is that traits can be similar based on shared ancestry, or because of convergent evolution due to similar selective pressures, or even be the result of neutral drift. Less weight can be given to trait similarity if species are closely related as measured by distance in a phylogeny, i.e. ‘phylogenetic signal’. A general method to account for this signal is to use phylogenetic generalized least squares (PGLS) [17]. By this method, we can determine whether two traits co-vary because of selection for fitness, independent of shared evolutionary history. The significance of the correlation is reduced for closely related species. Typically, many species are needed along with a phylogenetic tree with branch length estimates for robust determination of trait correlation. With the recent advent of genome-scale sequencing of >100 vertebrate species, the ability to correlate genomic features with physiological traits is now feasible. Thus, CpG density in gene promoters is a trait that can be selected and now measured in many species. Comparatively, the trait of maximum lifespan is also under selection and these traits can be correlated.

Genome-wide CGI density has been shown to correlate with body temperature and other traits, yet it does not correlate with lifespan [18]. Our own previous work finds that sequence feature density alone, independent of sequence context, can explain the evolution of features within the genome [19]. In this study, we link changes in CpG density that evolved over thousands of generations with changes in CpG methylation that occur within a single generation. Here we present the first identification of loci correlated to lifespan based on epigenetic features. We computationally analyzed the genomes of 131 mammal species (inclusive of 28 primates) within highly conserved promoter regions for the presence of CpG density correlated with lifespan, using data publically available from the AnAge database [20]. For both primates and mammals approximately 5% of gene promoters increase in CpG density along with lifespan.

## RESULTS

### Identification of homologous promoters across Mammalia

**Figure 1:**
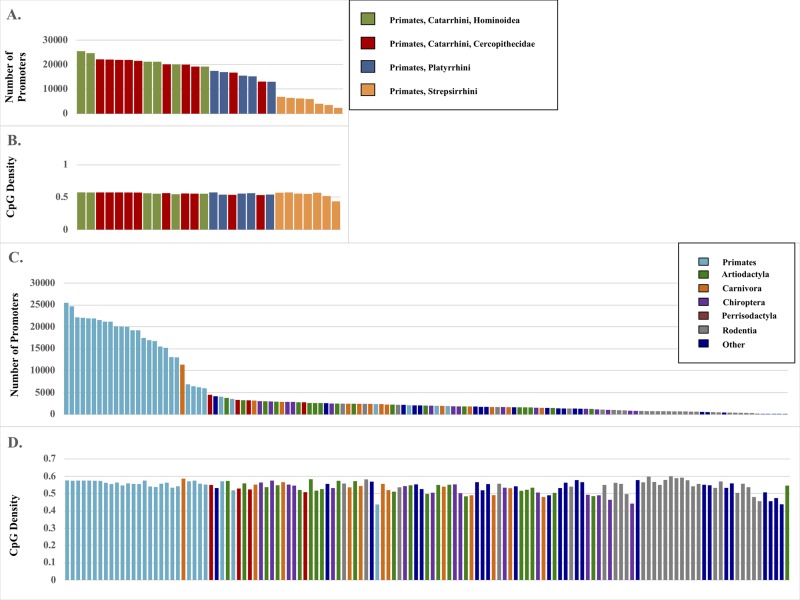
**Promoter matches by species and GC content.** (**A**) Total number of promoters identified by BLAST search of 28 primate genomes. (**B**) The associated GC content (%) of those promoter regions. (**C**) Total number of promoters identified from 131 primate genomes. (**D**) The associated GC content (%) of those promoter regions.

From the EPDnew database, 25,503 experimentally verified human promoter sequences were used as queries to BLAST genomes of 131 species of mammals across 23 orders, inclusive of 28 primates (Table 1). The query consisted of promoter sequence from -499 to +100 nt of sequence at each annotated human promoter as downloaded from the full EPDnew database. Best hit matches for each promoter were kept for each species. Species most genetically similar to human yielded a higher number of total >70% identity matches to human, as well as greater >95% and >99% matches (Figure 1 & Table 1). While the total number of promoters identified varied greatly across mammalian genomes across millions of years (MYA), from 24,686 in *Pan troglodytes* (chimp, 6 MYA since human common ancestor) to 3 in *Odocoileus virginianus* (white-tailed deer, 94 MYA), GC content remained consistent across species from 43.7% in *Daubentonia madagascariensis* (aye-aye, 73 MYA) to 60.0% in *Mus pahri* (Gairdner’s shrewmouse, 90 MYA) (Table S1). The total number of promoters identified in primate genomes ranged from 25,503 in human to 2,375 in aye-aye. Similarly to GC content in all mammals, primates remained consistent, from 57.6% in human to 43.7% in *D. madagascariensis*. Consistency was observed in total promoter number and density across primate lineages, with the most closely related species (Hominidae) displaying the most similarity to the human genome. Table S2 reports species level results.

**Table 1:** Summary list of taxon orders examined in this study. Order names are given with number of promoters matched to human promoters at various percent identity cutoffs. Table S2 contains a full list of taxa with the number of identified promoters in each species.

Order	No. Species	Promoters Identified with >=70% match	Promoters Identified with >=90% match	Promoters Identified with >=95% match	Promoters Identified with >=99% match
Afrosoricida	2	1882	170	22	0
Artiodactyla	23	50580	4941	618	22
Carnivora	12	35548	4669	826	161
Chiroptera	14	27485	2346	276	12
Cingulata	1	1730	162	18	0
Dasyuromorphia	1	51	7	2	0
Dermoptera	1	4159	410	47	1
Didelphimorphia	1	63	11	2	0
Diprotodontia	1	35	6	1	0
Eulypotyphla	3	2377	283	35	3
Hyracoidea	1	1306	101	14	1
Lagomorpha	1	1641	163	24	1
Macroscelidea	1	810	89	15	1
Monotremata	1	19	3	1	1
Perissodactyla	4	13807	1349	180	5
Pholidota	2	13826	279	36	2
Pilosa	1	2195	193	19	0
Primates	28	445897	346165	172222	47784
Proboscidea	1	2067	176	18	1
Rodentia	28	25060	2738	353	5
Scandentia	2	3121	362	41	2
Sirenia	1	2574	203	23	1
Tubulidentata	1	1811	170	20	1

**Figure 2:**
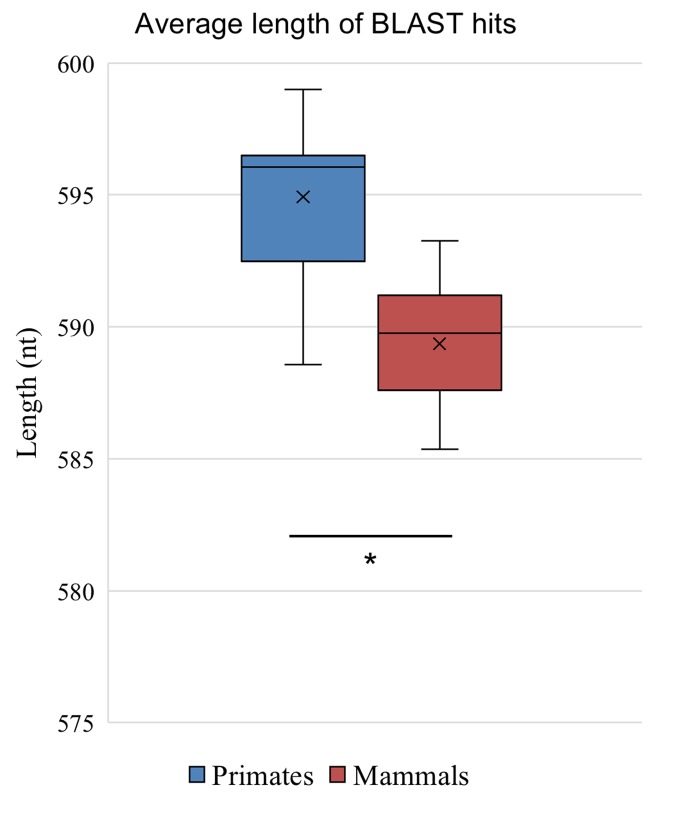
**Length of promoters.** The average length of BLAST detected promoters from the group of 28 primates and the set of 103 mammals excluding primates shows shorter length sequences in the mammalian group (p<0.0001).

In addition to identifying fewer matches at all levels of percentage identity in species more distantly related to humans, the matches were slightly shorter. Despite the large difference in the count of identified homologous promoters in species closely related to humans vs. more distantly related primates (e.g. 24,686 matches in chimp vs. 2375 in aye-aye), the length of the >70% matches remained in a tight range and these were used for all further analyses (Figure 2). When comparing primates with an average match length of 595 nt, there is only a small decrease in length of the matches identified in the mammal group when primates are excluded, at 589 nt average per match. This is due to the anthropocentric bias of our dataset, given the use of the *Homo sapiens* genome as the initial source of promoter data.

### Visualization of the best correlated promoters

**Figure 3:**
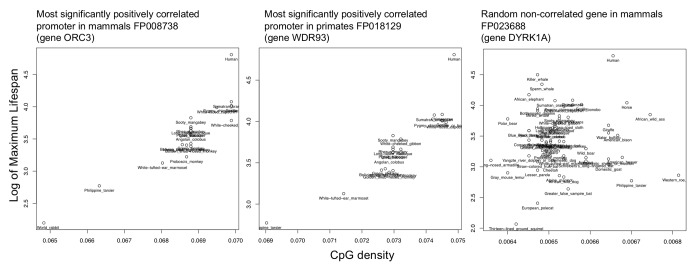
**Example correlations of top hit and random promoter.** Shown are the scatterplots of the log(max lifespan) vs. CpG density values for the most significantly correlated promoters from each of the mammalian and primate datasets as compared to a random non-correlated gene.

We performed a correlation analysis of the log(max lifespan) vs. CpG density values for promoters from each of the mammalian and primate datasets and compare to a random non-correlated gene (Figure 3). Each promoter was associated with a different number of species, assessed for significance adjusted to give a q-value (Table S3). Gene names were annotated for the nearest gene according to the EPDnew database.

### Primate results

**Figure 4:**
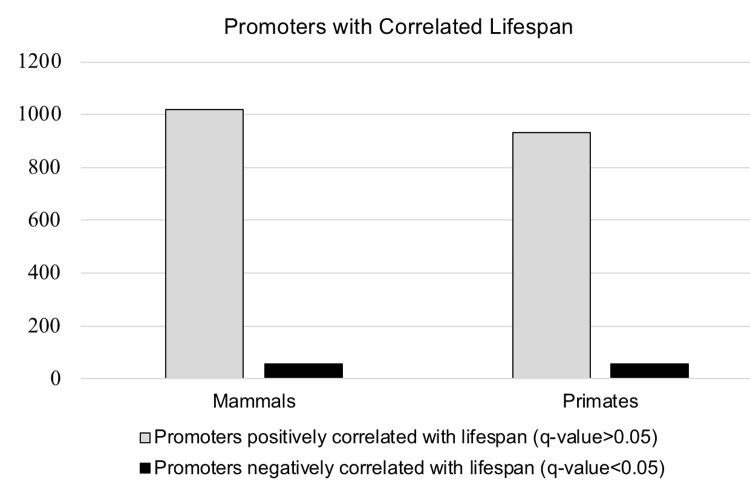
**Visualization of the data presented in** **Table 2****.** The number of promoters correlated with lifespan in the entire mammalian dataset, and in the primate dataset only.

A total of 987 promoters out of 25,503 had a significant correlation between increased CpG density and increased lifespan, of which 930 were positively correlated while only 57 were negatively correlated (q<0.05) (Figure 4 & Table 2). There was an average of 17.8 species present per promoter in the overall primate dataset, and 17.0 species present on average for promoters with q-value <0.05. Since multiple promoters can be annotated to the same gene, we checked for duplicate genes. The 930 positively correlated promoters corresponded to 912 unique gene annotations while all 57 of the negatively correlated promoters annotated as unique gene regions (Table S3).

**Table 2:** Number of genes positively (q-value >0.05) and negatively (q-value <0.05) correlated with lifespan in both the entire mammalian dataset (131 species), and the primate subset (28 species) only.

	**Mammals**	**Primates**
Promoters positively correlated with lifespan (q-value>0.05)	1020	930
Promoters negatively correlated with lifespan (q-value<0.05)	59	57
Total number of promoters with a significant q-value (>0.05)	1079	987

### Mammal results

Mirroring the primate results, a total of 1079 promoters out of 25,503 initially identified in the genome of *H. sapiens* had a significant positive correlation between increased CpG density and increased lifespan, 1020 were positively correlated while only 59 were negatively correlated (q<0.05) (Figure 4 & Table 2). There were an average of 24.0 species present per promoter overall, and 19.6 species for promoters with q-value <0.05. The 1020 positively correlated promoters were annotated to 999 unique genes, and all 59 negatively correlated promoters corresponded to unique genes (Table S3).

### Bias towards increasing CpG density with lifespan and conserved correlation

**Figure 5:**
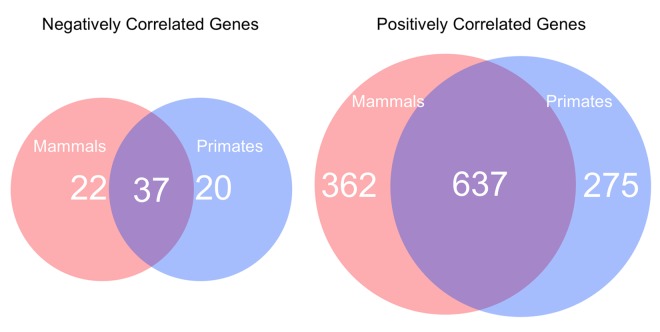
**The number of negatively and positively correlated lifespan-related genes in the whole mammalian dataset compared to those specific to primates.** Negatively correlated genes and positively correlated genes.

Our analyses of both the primate subset and the whole mammalian dataset both yielded a ~95% skew toward positive correlation of the identified promoters with species lifespan (p<0.001). Of the positively correlated loci identified, 637 were shared between the primates-only dataset and the whole mammalian dataset (Figure 5). A total of 275 loci were specific to the 28 primate species surveyed, while 362 were found only in non-primate mammal species surveyed. Of the negatively correlated loci identified, 37 were shared between the primates-only dataset and the whole mammalian dataset. 20 loci were specific to the primate dataset, while 22 loci were found in other mammalian species but were not present in primates.

### Gene set enrichment

Each set of genes, positively and negatively correlated, from both primate and mammal datasets was examined for enrichment in biological processes using the EnrichR tool [16]. When sorted by gene ontology categories, GO Cellular Component, GO Biological Process, and GO Molecular Function 2017b, no significant enrichment was observed for any group of genes in any category (Table S4).

## DISCUSSION

Increasing evidence has linked quantifiable phenotypic traits to DNA methylation status in regions around active genes. Over the long term, regions with low CpG density undergo mutation at a much more rapid rate than highly CpG-dense regions [21]. Base excision repair pathways have been shown to correlate with longer lived species and may influence the rate of deamination induced CpG mutations [22]. However, here we are most interested in the evolution and the gradation of underlying CpG density of promoter regions as a consequence of natural selection in the context of rapid mutation of CpG sites across species. We shed light on the question of how the evolution of CpG density correlates with a physiological trait. Our hypothesis is that greater CpG density in some genes gives more buffering capacity to absorb age-related changes in methylation without negatively affecting their expression.

Within the lifespan of a single individual, studies have linked the hypermethylation of CpG sites to aging [23]. This can be measured. Horvath et al. have developed an epigenetic “clock” capable of predicting DNA methylation age across a wide variety of tissue samples (both healthy and cancerous) with a high degree of accuracy based on 353 CpG sites [15]. A comparable multi-tissue murine clock based on 329 CpG sites has also been demonstrated [24]. While these studies identify specific CpG sites whose methylation changes with age, they do not identify genes that have likely been under selection to generate high CpG density to allow longer lifespans. We believe the genes identified here have been under selective pressure in concert with the evolution of slower aging or longer lifespan among mammals.

We broke our dataset into two groups, mammals and primates, since we expect that the collection of genes that have evolved to affect lifespan is likely clade-specific. In other words, genes under selection pressure that influence lifespan, are more likely to be shared in closely related species than the genes underlying long lifespan in distantly related species. However, CpG density of the promoter complement of each species remains in a tight range regardless of the number of promoters matched in each species, indicating no bias towards or against promoters by GC density. The pool of promoters is based on conservation to the human sequence, biasing our results on genes that influence longevity that exist in the human.

Within primates, the maximum lifespan of humans is between 90-100 years, with relatively rare outliers surviving ~10-20 years beyond this [20,25]. Lifespan in non-human primates varies by species and lifestyle. A closely related great ape, the western lowland gorilla (*Gorilla gorilla*) displays similar maximum lifespan to the chimpanzee, with the oldest verified captive animals living to ~60 and wild individuals living to at least age 43 [20,26]. More distantly related apes such as gibbons of the genus *Hylobates* display some variation in maximum lifespan ranging from 37 years (*Hylobates klossii*) to 60 years (*Hylobates muelleri*). Old and New World monkeys can survive for ~40 years or more in captivity. Captive Strepshirrine primates such as lemurs and lorises can live into their 20s and 30s, while much shorter lives are common in the wild [20]. Ultimately, the genes that have evolved to enable the generally long lifespans in primates likely overlap highly between these species due to shared ancestry. Our primate results indicate about 5% of genes have CpG density in promoters under selection for increasing CpG density. Remarkably in 95% of these significantly correlated genes, CpG density increased concomitantly with lifespan.

Outside of the Order Primates, lifespan in the mammalian radiation is highly variable. Some species, African elephants and orcas for example, equal or exceed the maximum observed lifespan of *H. sapiens* and other great apes. Other mammals greatly exceed observed primate lifespans. Bowhead whales have been demonstrated to more than double the maximum human lifespan [20]. Long lifespan has evolved at least 4 separate times in rodents and in closely related lagomorphs (i.e. varmints) [27]. Surprisingly, the same number of genes, about 5% of the total mammalian complement of ~20,000 genes, show increasing CpG density with lifespan in the mammal dataset. Again 94% of these had a positive correlation. We found that the positively correlated genes largely, but not completely, overlapped between the datasets. This fits with our hypothesis, that evolution of long lifespan is driven by different genes in distantly related species, and at the same time, convergent evolution has resulted in many of the same core genes being selected as well.

There are some potential sources of error in our study. Because shared ancestry can artificially increase seeming correlation between traits, we corrected for this effect by using phylogenetic generalized least squares. Most loci identified here had less than a dozen species represented per gene, so results could be improved by better sequencing and alignment between species. To correct for multiple comparisons, using over 24,000 loci, we adjusted our p-values using Benjamini-Hochberg correction. Given the nature of the large dataset and analysis here, potential for false positives is high. Our analysis pipeline has no bias in directionality of correlated genes. A gene with high and significant negative correlation is just as likely to be detected as one with positive correlation. Despite this, the ~95% bias towards positively correlated genes strongly indicates a real biological signal underlies our identified gene sets. A true source of bias is that our study is inherently human-centric in that human promoters were considered as the reference sequence. As a result of this methodology, identified promoters whose CpG density correlates with lifespan must be present in the human genome. Consequently, our analysis surely misses loci in non-human species that are tied to lifespan.

Since aging is not under direct selection, it is recognized that genes affecting these traits are not likely to be tied to any particular biological pathway, inconsistent with the idea of aging as a programmed phenomenon [28]. Our EnrichR analyses are consistent with this hypothesis, finding no enriched pathways among our gene sets.

As further genomes become available and life history traits are recorded for an increasing number of species, correlations between additional physiological traits and gene features becomes more feasible. Regarding maximum lifespan and CpG density, this study provides strong evidence that CpG density in some genes likely provides buffering capacity, thereby linking organismal aging to evolution of mammalian genomes.

## METHODS

### Identification of conserved promoters

The genomes of 131 mammals (including 28 primate species) were downloaded from NCBI as unmasked fasta files (Table 1). Human promoter sequences were used to query each genome via BLAST v2.6.0+ [29]. BLAST parameters were, “blastn -query <human promoters.fasta> -db <target genome> -task megablast -max_hsps 1 -outfmt "6 qseqid qlen qstart qend sacc sstart send evalue bitscore length pident qcovhsp qseq sseq" -culling_limit 1 > <output file>”.

Experimentally validated human promoters, EPDnew (n = 25503), were downloaded from the Eukaryotic Promoter Database website, comprising of -499 to +100 nt of sequence encompassing each promoter [30]. The EPD consortium has validated these by high-throughput transcription start site mapping. BLAST was run locally with the promoter list against each species for each promoter and only a single top hit with >70% identity was kept per species. Additional filters of >95% and >99% identity were applied to determine conservation across species as divergence time receded.

### CpG frequency calculation

CpG frequency was calculated for each BLAST hit with the CpG_calculator.pl tool from BioToolBox (https://github.com/tjparnell/biotoolbox). Results were merged with BLAST output via custom scripts. Each promoter was assigned to an individual file containing all species’ BLAST hits and CpG count data.

### Phylogenetic tree and physiological traits

The Animal Ageing and Longevity Database (AnAge), release 13, was downloaded, including physiological traits for infant mortality rate (IMR), mortality rate doubling time (MRDT), maximum longevity (i.e. the oldest verified lifespan of an individual), female and male sexual maturity, gestation time, weaning length, litter size, litters per year, litter interval, birth weight, wean weight, adult weight, postnatal growth rate [20]. Maximum lifespan in years was natural log transformed. The tree for primates was downloaded from the 10ktrees project (http://10ktrees.fas.harvard.edu), order Primates version 3, as a consensus tree with chronogram branch lengths and contained 301 species [31]. The tree for mammals was derived from the Bininda-Emonds supertree of mammals containing 4510 species [32].

### Maximum lifespan

For the species in this analysis, maximum lifespan was obtained from the curated AnAge database which contained data for 109 of our species. The mean maximum lifespan was 28 years for 109 mammal species and 37.7 years for the 28 primate species. For further data analysis, the log of the maximum lifespan was used.

For humans, the maximum lifespan was set at 90 years to account for the huge sample size in this species, resulting in a maximum lifespan that is not comparable to species for which the sample size is much smaller. The maximum verified age reached by a human is 122, but such outliers are not representative of the typical human lifespan.

Some animals were not represented in the AnAge database so physiological data were inferred from closely related species and literature reports. The AnAge database entries were corrected to reflect either current species nomenclature or to substitute the nearest related species. In calculating the average length of BLAST matches, *Odobenus rosmarus* (walrus) was removed as an extreme outlier. Divergence times from the human lineage were taken from timetree.org.

### Phylogenetic least squares analysis

PGLS analysis was implemented in R version 3.3.3 using the packages caper, version 0.5.2, and APE [33]. The model used was, “model.pgls<-pgls(log(max_lifespan_yrs) ~ cpg_freq, data = combodata, lambda='ML')”. Various body traits correlate with lifespan, such as developmental times and body weight [34,35], however these parameters were not applied here because for any given gene the number of species was very low. The paucity of data in AnAge for these traits restricts the species retained to a number too small for analysis. No best fit model was applied because the combination of predictor variables best fitting the dependent variable would be different for each of the 25,503 loci, and secondly, we are not interested in the overall best fit of the explanatory variables, but primarily the contribution of the number of CpG sites for maximum lifespan. Pearson correlations were calculated and tested significance was reported as p-value. Benjamani-Hochberg adjustment was applied to the resulting p-values to correct for multiple testing, yielding q-values, which were used for further analysis.

### Gene set enrichment analysis

Gene lists of significant q-values were submitted to EnrichR for gene set enrichment analyses. Genes were sorted by ontology categories, GO Cellular Component 2017b, GO Biological Process 2017b, and GO Molecular Function 2017b and adjusted p-value was used to determine significant enrichment [16].

## Supplementary Material

Supplementary Table S1

Supplementary Table S2

Supplementary Table S3

Supplementary Table S4
