# Supplementary material for: The evolution of CpG density and lifespan in conserved primate and mammalian promoters
Source: Aging (Albany NY). 2018 Apr 14;10(4):561–72. doi: 10.18632/aging.101413 (PMC5940106; doi:10.18632/aging.101413)
Supplement: Supplementary Table S1 [file aging-10-101413-s001.pdf]

Primate Species	Number of promoters	Start length	End length	Fraction GC	Observed CpGs	Expected CpGs	Obs/Exp
Homo_sapiens	25496	1	15297555	57.60%	867252	1266419	0.685
Pan_troglodytes	24686	1	14801424	57.40%	832579	1219389	0.683
Cercocebus_atys	22165	1	13256651	57.50%	751908	1097305	0.685
Chlorocebus_sabaeus	22072	1	13200850	57.50%	745173	1089799	0.684
Macaca_nemestrina	21945	1	13124762	57.50%	741106	1084191	0.684
Macaca_mulatta	21918	1	13107550	57.40%	735277	1078297	0.682
Papio_anubis	21554	1	12890519	57.30%	717992	1057687	0.679
Pongo_abelii	21190	1	12690956	56.20%	661363	1002220	0.66
Gorilla_gorilla	21187	1	12698925	55.50%	623642	978019	0.638
Rhinopithecus_bieti	20124	1	12032608	56.40%	627803	956666	0.656
Pan_paniscus	20084	1	12038908	54.70%	550390	899999	0.612
Macaca_fascicularis	20017	1	11971569	55.90%	605107	936376	0.646
Rhinopithecus_roxellana	19222	1	11493604	55.60%	567183	889077	0.638
Nomascus_leucogenys	19213	1	11502300	55.50%	573346	886402	0.647
Cebus_capucinus	17455	1	10410861	57.50%	591526	861316	0.687
Colobus_angolensis	16946	1	10132443	54.10%	440038	740859	0.594
Mandrillus_leucophaeus	16751	1	10018046	53.80%	423782	724790	0.585
Aotus_nancymaae	15514	1	9254069	55.70%	460858	717208	0.643
Callithrix_jacchus	15224	1	9074085	56.30%	476796	719963	0.662
Nasalis_larvatus	13093	1	7824496	53.40%	333311	558296	0.597
Saimiri_boliviensis	13024	1	7762913	54.20%	339848	569901	0.596
Propithecus_coquereli	6879	1	4082077	57.10%	232330	332433	0.699
Microcebus_murinus	6434	1	3815374	57.50%	224108	315059	0.711
Eulemur_flavifrons	6205	1	3681076	55.70%	191065	285749	0.669
Eulemur_macaco	5980	1	3546861	55.20%	177456	270121	0.657
Otolemur_garnettii	4058	1	2403176	57.10%	135720	195641	0.694
Carlito_syrichta	3549	1	2102866	51.90%	80160	141735	0.566
Daubentonia_madagasca	2375	1	1409230	43.70%	20863	67192	0.31

Mammalian Species	Number of promoters	Start length	End length	Fraction GC	Observed CpGs	Expected CpGs	Obs/Exp
Homo_sapiens	25496	1	15297555	57.6%	867252	1266419	0.685
Pan_troglodytes	24686	1	14801424	57.4%	832579	1219389	0.683
Cercocebus_atys	22165	1	13256651	57.5%	751908	1097305	0.685
Chlorocebus_sabaeu	22072	1	13200850	57.5%	745173	1089799	0.684
Macaca_nemestrina	21945	1	13124762	57.5%	741106	1084191	0.684
Macaca_mulatta	21918	1	13107550	57.4%	735277	1078297	0.682
Papio_anubis	21554	1	12890519	57.3%	717992	1057687	0.679
Pongo_abelii	21190	1	12690956	56.2%	661363	1002220	0.66
Gorilla_gorilla	21187	1	12698925	55.5%	623642	978019	0.638
Rhinopithecus_bieti	20124	1	12032608	56.4%	627803	956666	0.656
Pan_paniscus	20084	1	12038908	54.7%	550390	899999	0.612
Macaca_fascicularis	20017	1	11971569	55.9%	605107	936376	0.646

Rhinopithecus_roxella	19222	1	11493604	55.6%	567183	889077	0.638
Nomascus_leucogeny	19213	1	11502300	55.5%	573346	886402	0.647
Cebus_capucinus	17455	1	10410861	57.5%	591526	861316	0.687
Colobus_angolensis	16946	1	10132443	54.1%	440038	740859	0.594
Mandrillus_leucophae	16751	1	10018046	53.8%	423782	724790	0.585
Aotus_nancymaae	15514	1	9254069	55.7%	460858	717208	0.643
Callithrix_jacchus	15224	1	9074085	56.3%	476796	719963	0.662
Nasalis_larvatus	13093	1	7824496	53.4%	333311	558296	0.597
Saimiri_boliviensis	13024	1	7762913	54.2%	339848	569901	0.596
Odobenus_rosmarus	11373	1	4651043	58.6%	293246	398984	0.735
Propithecus_coquere	6879	1	4082077	57.1%	232330	332433	0.699
Microcebus_murinus	6434	1	3815374	57.5%	224108	315059	0.711
Eulemur_flavifrons	6205	1	3681076	55.7%	191065	285749	0.669
Eulemur_macaco	5980	1	3546861	55.2%	177456	270121	0.657
Ceratotherium_simun	4488	1	2657807	55.0%	132259	200937	0.658
Galeopterus_variegat	4159	1	2464370	53.2%	105406	174328	0.605
Otolemur_garnettii	4058	1	2403176	57.1%	135720	195641	0.694
Orcinus_orca	3774	1	2235559	57.3%	131331	183277	0.717
Carlito_syrichta	3549	1	2102866	51.9%	80160	141735	0.566
Equus_asinus	3317	1	1963923	52.9%	85102	137535	0.619
Lipotes_vexillifer	3249	1	1923448	55.9%	104532	150084	0.696
Equus_caballus	3228	1	1911339	52.4%	77747	131066	0.593
Leptonychotes_wedd	3169	1	1875944	55.2%	96163	143095	0.672
Hipposideros_armige	3034	1	1795506	56.4%	98921	142876	0.692
Physeter_macroceph	3009	1	1780995	53.7%	81193	128216	0.633
Rousettus_egyptiacus	2968	1	1756048	57.5%	105388	145138	0.726
Tursiops_truncatus	2918	1	1727528	54.8%	86670	129588	0.669
Panthera_pardus	2870	1	1698851	56.6%	97339	135918	0.716
Pteropus_vampyrus	2868	1	1697166	55.2%	86335	129347	0.667
Rhinolophus_sinicus	2866	1	1696365	54.6%	83643	126308	0.662
Balaenoptera_acutoro	2780	1	1646281	52.1%	66837	111831	0.598
Equus_przewalskii	2774	1	1641951	50.8%	59186	105750	0.56
Capra_hircus	2623	1	1551477	58.3%	97390	131577	0.74
Balaenoptera_bonaer	2603	1	1540768	51.7%	60493	102863	0.588
Vicugna_pacos	2600	1	1538298	52.6%	63899	106343	0.601
Trichechus_manatus	2574	1	1521901	55.6%	79040	117702	0.672
Pteropus_alecto	2493	1	1474906	53.2%	65237	104467	0.624
Giraffa_camelopardal	2481	1	1467277	57.4%	88304	120817	0.731
Ictidomys_tridecemlin	2469	1	1460452	55.8%	76517	113629	0.673
Felis_catus	2455	1	1452944	53.6%	66081	104212	0.634
Okapia_johnstoni	2445	1	1445787	57.2%	85406	118349	0.722
Ailurus_fulgens	2434	1	1440366	54.4%	70358	106446	0.661
Castor_canadensis	2426	1	1435538	58.2%	88404	121581	0.727
Sus_scrofa	2414	1	1428346	56.9%	81841	115485	0.709
Daubentonia_madaga	2375	1	1409230	43.7%	20863	67192	0.31
Mustela_putorius	2363	1	1397675	55.6%	73517	107822	0.682

Ailuropoda_melanole	2255	1	1334828	52.0%	54122	90220	0.6
Camelus_bactrianus	2249	1	1330529	51.1%	48592	86680	0.561
Marmota_marmota	2198	1	1299712	53.6%	58775	93254	0.63
Choloepus_hoffmann	2195	1	1297799	54.3%	63178	95496	0.662
Bubalus_bubalis	2093	1	1237456	54.8%	62190	93023	0.669
Loxodonta_africana	2067	1	1222770	55.3%	61962	93366	0.664
Manis_javalinca	2057	1	1215527	52.6%	52192	84016	0.621
Camelus_dromedariu	2022	1	1195921	49.8%	39685	74129	0.535
Rhinolophus_ferrume	2003	1	1184704	50.5%	42544	75641	0.562
Bos_indicus	1975	1	1167755	55.0%	59150	88381	0.669
Canis_familiaris	1950	1	1153958	54.0%	54781	84067	0.652
Ovis_aries	1929	1	1140450	55.1%	59890	86424	0.693
Eptesicus_fuscus	1860	1	1099638	55.3%	55903	84035	0.665
Eidolon_helvum	1836	1	1084983	50.2%	38158	68278	0.559
Camelus_ferus	1831	1	1082989	48.4%	31023	63433	0.489
Ursus_maritimus	1819	1	1076832	49.0%	33931	64631	0.525
Orycteropus_afer	1811	1	1070604	56.6%	59118	85630	0.69
Tupaia_chinensis	1731	1	1023212	51.9%	40113	68900	0.582
Dasypus_novemcinct	1730	1	1023204	55.5%	55176	78762	0.701
Panthera_tigris	1692	1	1001411	49.1%	31263	60296	0.518
Heterocephalus_glab	1691	1	1000227	55.7%	53939	77606	0.695
Myotis_lucifugus	1691	1	999700	53.4%	44922	71222	0.631
Lycaon_pictus	1665	1	985036	53.1%	44062	69476	0.634
Oryctolagus_cuniculu	1641	1	971029	54.2%	48631	71367	0.681
Bos_mutus	1628	1	962414	51.6%	37956	64029	0.593
Bison_bison	1617	1	955763	52.2%	40485	65111	0.622
Capreolus_capreolus	1602	1	946654	53.4%	43775	67577	0.648
Miniopterus_natalens	1531	1	905341	50.6%	32564	57954	0.562
Acinonyx_jubatus	1503	1	889227	48.0%	25301	51247	0.494
Manis_pentadactyla	1495	1	883897	49.0%	26971	52927	0.51
Pantholops_hodgson	1479	1	874299	50.4%	31228	55401	0.564
Tupaia_belangeri	1390	1	821406	53.2%	36736	58134	0.632
Condylura_cristata	1359	1	803576	56.3%	43715	63693	0.686
Chinchilla_lanigera	1352	1	799903	54.0%	37203	58285	0.638
Chrysochloris_asiatic	1346	1	795919	57.8%	47824	66402	0.72
Procavia_capensis	1306	1	771482	56.6%	42882	61750	0.694
Myotis_brandti	1296	1	766681	49.3%	23187	46635	0.497
Capra_aegagrus	1256	1	742190	48.5%	22125	43688	0.506
Myotis_mystacinus	1148	1	678846	49.0%	20407	40764	0.501
Cavia_porcellus	1107	1	654371	55.0%	33014	49432	0.668
Pteronotus_parnellii	1050	1	620813	46.4%	15484	33419	0.463
Octodon_degus	1039	1	614253	56.2%	33005	48523	0.68
Nannospalax_galili	954	1	563876	55.6%	28625	43594	0.657
Cryptomys_damarens	924	1	546420	49.7%	18301	33740	0.542
Megaderma_lyra	841	1	496981	44.2%	9147	24232	0.377
Elephantulus_edward	810	1	479238	57.8%	27814	39975	0.696

Ochotona_princeps	796	1	470360	56.5%	26631	37563	0.709
Mus_caroli	751	1	444086	59.8%	29447	39675	0.742
Peromyscus_maniculatus	742	1	438226	56.7%	23554	35152	0.67
Dipodomys_ordii	735	1	434259	55.0%	21460	32771	0.655
Microtus_ochrogaster	733	1	433369	57.9%	25459	36333	0.701
Mus_pahari	718	1	424567	60.0%	28412	38187	0.744
Rattus_norvegicus	698	1	412597	58.9%	25940	35823	0.724
Mus_spretus	695	1	410995	59.2%	26455	35990	0.735
Mesocricetus_auratus	692	1	409148	57.7%	24024	34048	0.706
Jaculus_jaculus	640	1	377717	54.2%	17350	27677	0.627
Neotoma_lepida	635	1	374914	55.6%	19468	28941	0.673
Erinaceus_europaeus	581	1	343212	55.1%	17767	26066	0.682
Echinops_telfairi	536	1	316674	54.8%	15880	23778	0.668
Ellobius_talpinus	534	1	315384	53.3%	13574	22354	0.607
Myodes_glareolus	484	1	285799	57.0%	16053	23178	0.693
Sorex_araneus	437	1	258509	53.3%	11729	18367	0.639
Microtus_agrestis	430	1	253965	55.9%	13318	19832	0.672
Cricetulus_barabensis	408	1	241384	50.4%	8171	15345	0.532
Apodemus_sylvaticus	374	1	220795	55.7%	11678	17149	0.681
Phodopus_sungorus	347	1	205131	53.7%	9469	14771	0.641
Ellobius_lutescens	301	1	177567	48.0%	4657	10235	0.455
Cavia_aperea	187	1	110385	45.6%	1914	5743	0.333
Monodelphis_domest	63	1	37184	50.7%	1484	2392	0.62
Sarcophilus_harrisii	51	1	30219	45.6%	703	1571	0.447
Macropus_eugenii	35	1	20725	47.4%	595	1162	0.512
Ornithorhynchus_ana	19	1	11204	43.8%	240	537	0.447
Odocoileus_virginianu	3	1	1744	54.6%	85	130	0.654
