# Supplementary material for: The evolution of CpG density and lifespan in conserved primate and mammalian promoters
Source: Aging (Albany NY). 2018 Apr 14;10(4):561–72. doi: 10.18632/aging.101413 (PMC5940106; doi:10.18632/aging.101413)
Supplement: Supplementary Table S2 [file aging-10-101413-s002.pdf]

Order	Species	Promoters Identified > 70% match	Promoters Identified >= 90% match	Promoters Identified >= 95% match	Promoters Identified >= 99% match
**Afrosoricida**					
	Chrysochloris asiatica	1346	126	17	0
	Echinops telfairi	536	44	5	0
**Artiodactyla (Cetacea)**					
	Orcinus orca	3774	411	49	1
	Physeter macrocephalus	3009	267	31	1
	Tursiops truncatus	2918	269	30	1
	Balaenoptera acutorostrata	2780	235	33	1
	Lipotes vexillifer	3249	302	35	1
	Balaenoptera bonaerensis	2603	215	26	1
**Artiodactyla (Artiofabula)**					
	Sus scrofa	2414	264	36	1
**Artiodactyla (Ruminantiamorpha)**					
	Okapia johnstoni	2445	255	34	1
	Giraffa camelopardalis	2481	269	32	1
	Capreolus capreolus	1602	148	16	1
	Odocoileus virginianus	3	0	0	0
	Bos indicus	1975	197	23	1
	Bos mutus	1628	160	23	1
	Bison bison	1617	164	24	1
	Pantholops hodgsonii	1479	138	18	1
	Capra hircus	2623	307	41	1
	Capra aegagrus	1256	115	16	1
	Ovis aries	1929	179	22	1
	Bubalus bubalis	2093	205	24	1
**Artiodactyla (Tylopoda)**					
	Camelus bactrianus	2249	204	24	1
	Camelus dromedarius	2022	197	30	1
	Camelus ferus	1831	181	23	1
	Vicugna pacos	2600	259	28	1
**Carnivora**					
	Panthera pardus	2870	335	41	1
	Felis catus	2455	282	34	1
	Canis familiaris	1950	250	35	0
	Ursus maritimus	1819	207	33	1
	Panthera tigris	1692	198	23	1
	Leptonychotes weddellii	3169	345	39	1

	Ailurus fulgens	2434	262	32	1
	Mustela putorius	2363	279	37	1
	Ailuropoda melanoleuca	2255	252	31	1
	Lycaon pictus	1665	204	27	0
	Acinonyx jubatus	1503	175	22	1
**Chiroptera**					
	Hipposideros armiger	3034	250	25	1
	Rousettus egyptiacus	2968	294	38	1
	Pteropus vampyrus	2868	282	37	1
	Rhinolophus sinicus	2866	240	23	1
	Pteropus alecto	2493	213	26	1
	Rhinolophus ferrumequinum	2003	151	18	1
	Eptesicus fuscus	1860	148	20	1
	Eidolon helvum	1836	145	19	1
	Myotis lucifugus	1691	134	14	1
	Miniopterus natalensis	1531	114	13	0
	Myotis brandti	1296	117	12	1
	Myotis mystacinus	1148	95	8	1
	Pteronotus parnellii	1050	94	12	1
	Megaderma lyra	841	69	11	0
**Cingulata**					
	Dasypus novemcinctus	1730	162	18	0
**Dasyuromorphia**					
	Sarcophilus harrisii	51	7	2	0
**Dermoptera**					
	Galeopterus variegatus	4159	410	47	1
**Didelphimorphia**					
	Monodelphis domestica	63	11	2	0
**Diprotodontia**					
	Macropus eugenii	35	6	1	0
**Eulypotyphla**					
	Sorex araneus	437	59	7	1
	Condylura cristata	1359	160	21	1
	Erinaceus europaeus	581	64	7	0
**Hyracoidea**					
	Procavia capensis	1306	101	14	1
**Lagomorpha**					
	Oryctolagus cuniculus	1641	163	24	1

**Macroscelidea**					
	Elephantulus edwardii	810	89	15	1
**Monotremata**					
	Ornithorhynchus anatinus	19	3	1	1
**Perissodactyla**					
	Ceratotherium simum	4488	479	58	2
	Equus asinus	3317	306	43	1
	Equus caballus	3228	313	43	1
	Equus przewalskii	2774	251	36	1
**Pholidota**					
	Manis javalinca	2057	157	19	1
	Manis pentadactyla	1495	122	17	1
**Pilosa**					
	Choloepus hoffmanni	2195	193	19	0
**Primates**					
	Homo sapiens	25496	25495	25495	25472
	Pan troglodytes	24686	24629	23997	9441
	Cercocebus atys	22165	19055	5376	61
	Chlorocebus sabaeus	22072	19078	5207	52
	Macaca nemestrina	21945	18839	5196	51
	Macaca mulatta	21918	18695	5408	49
	Papio anubis	21554	18741	5238	49
	Pongo abelii	21190	20953	16342	320
	Gorilla gorilla	21187	21115	20380	4257
	Rhinopithecus bieti	20124	16899	4112	23
	Pan paniscus	20084	20009	19593	7627
	Macaca fascicularis	20017	17564	4959	44
	Rhinopithecus roxellana	19222	16567	4139	34
	Nomascus leucogenys	19213	18805	12748	176
	Cebus capucinus	17455	7060	828	10
	Colobus angolensis	16946	14626	3727	27
	Mandrillus leucophaeus	16751	14758	4107	39
	Aotus nancymaae	15514	6921	750	7
	Callithrix jacchus	15224	5495	549	7
	Nasalis larvatus	13093	11343	2910	24
	Saimiri boliviensis	13024	4949	552	4
	Odobenus rosmarus	11373	1880	472	152
	Propithecus coquereli	6879	950	134	4
	Microcebus murinus	6434	864	117	1
	Eulemur flavifrons	6205	793	103	1
	Eulemur macaco	5980	722	90	1

	Otolemur garnettii	4058	470	67	1
	Daubentonia madagascarien	2375	338	51	1
	Carlito syrichta	3549	432	47	1
**Proboscidea**					
	Loxodonta africana	2067	176	18	1
**Rodentia**					
	Mus caroli	751	86	11	0
	Mus pahari	718	83	11	0
	Rattus norvegicus	698	74	10	0
	Mus spretus	695	76	10	0
	Apodemus sylvaticus	374	36	3	0
	Castor canadensis	2426	292	32	1
	Cavia porcellus	1107	128	16	0
	Cavia aperea	187	33	5	0
	Chinchilla lanigera	1352	156	25	0
	Cricetulus griseus	408	46	5	0
	Dipodomys ordii	735	74	15	1
	Ellobius talpinus	534	56	6	0
	Ellobius lutescens	301	33	4	0
	Heterocephalus glaber	1691	162	23	0
	Ictidomys tridecemlineatus	2469	297	41	1
	Jaculus jaculus	640	56	7	0
	Marmota marmota	2198	226	35	0
	Mesocricetus auratus	692	77	9	0
	Microtus ochrogaster	733	81	7	0
	Microtus agrestis	430	45	4	0
	Myodes glareolus	484	46	3	0
	Nannospalax galili	954	92	10	0
	Neotoma lepida	635	69	7	0
	Peromyscus maniculatus	742	81	9	0
	Phodopus sungorus	347	37	3	0
	Octodon degus	1039	116	16	0
	Cryptomys damarensis	924	92	15	1
	Ochotona princeps	796	88	11	1
**Scandentia**					
	Tupaia chinensis	1731	200	24	1
	Tupaia belangeri	1390	162	17	1
**Sirenia**					
	Trichechus manatus	2574	203	23	1
**Tubulidentata**					
	Orycteropus afer	1811	170	20	1
